# Outcomes of robot-assisted versus video-assisted mediastinal mass resection during the initial learning curve

**DOI:** 10.1007/s11701-024-01828-7

**Published:** 2024-02-17

**Authors:** Chengwen Zheng, Yong Ge, Tianyue Ma, Jiajian Pan, Xueqiu Zhang, Teng Sun, Shoujie Feng, Hao Zhang

**Affiliations:** 1 Thoracic Surgery Laboratory, Xuzhou Medical University, Xuzhou, 221004 Jiangsu China; 2 Department of Thoracic Surgery, Affiliated Hospital of Xuzhou Medical University, Xuzhou, 221003 Jiangsu China

**Keywords:** Mediastinal mass, Robot-assisted thoracoscopic surgery, Thoracoscopy, Learning curve

## Abstract

To compare the learning curve of mediastinal mass resection between robot-assisted surgery and thoracoscopic surgery. Retrospective perioperative data were collected from 160 mediastinal mass resection cases. Data included 80 initial consecutive video-assisted thoracoscopic surgery (VATS) resection cases performed from February 2018 to February 2020 and 80 initial consecutive robotic-assisted thoracic surgery (RATS) resection cases performed from March 2020 to March 2023. All cases were operated on by a thoracic surgeon. The clinical characteristics and perioperative outcomes of the two groups were compared. The operation time in both the RATS group and VATS group was analyzed using the cumulative sum (CUSUM) method. Based on this method, the learning curves of both groups were divided into a learning period and mastery period. The VATS group and the RATS group crossed the inflection point in the 27th and 21st case, respectively. Subsequently, we found that the learning period was longer than the mastery period with statistically significant differences in terms of the operating time, and postoperative hospital stay in the VATS group and the RATS group. A certain amount of VATS experience can shorten the learning curve for RATS.

## Introduction

The mediastinum within the thoracic cavity includes the heart, macrovascular structures, trachea, esophagus, thymus, lymphatics, and nerves [1]. Consequently, the origin and location of mediastinal masses can be intricate and varied. The masses can be classified into neoplastic and non-neoplastic types. Neoplastic masses include thymoma, neuroendocrine tumors, malignant lymphomas, germ-cell tumors, thymolipoma, and metastases. In contrast, non-neoplastic lesions include intrathoracic goiters, thymic cysts, and aortic aneurysms. If surgery is a possibility, it is advisable to consider it actively as treatment for most mediastinal masses, regardless of their tumorigenic or non-tumorigenic nature [2, 3].

The video-assisted thoracoscopic surgery (VATS) technique is routinely performed for resection of mediastinal masses. Although patients undergoing thoracoscopic surgery achieve a similar prognosis compared to conventional open surgery, they experience a significantly lower rate of postoperative complications, hospitalization time, lower postoperative pain, and lesser blood loss [4–7]. In the past decade, robotic systems have been increasingly used in the field of surgery. High-definition 3-dimensional (3D) views, flexible robotic arms, and the ability to filter hand tremor make robotic systems ideal for surgeons to perform complex operations in confined spaces [8]. Li et al. reported that robotic-assisted thoracic surgery (RATS) technique for mediastinal mass resection was superior to VATS in terms of surgical blood loss and postoperative hospitalization time [9]. However, to the best of our knowledge, no study has independently compared the time required to skillfully perform mediastinal mass resection with VATS and RATS.

Surgical innovations often improve surgical outcomes and can reduce associated complications [10]. However, after implementing a new procedure, surgeons, anesthesiologists, and the nursing team must incorporate the new technique into their practice, which may help impact the surgery outcomes positively. The process of implementing new surgical techniques must, therefore, include a learning curve based on objective, measurable outcomes. Most thoracic surgeons performing RATS for mediastinal mass resection are experts in VATS for mediastinal mass resection before practicing robotic surgery. However, no study has independently compared the learning curves of the two procedures. Our hypothesis is that the learning curve for RATS may be shorter for a skilled VATS surgeon with regard to mediastinal mass resection. In this study, we investigated the effect of thoracoscopic surgical experience on the learning process of robotic surgery for mediastinal mass resection by retrospectively summarizing case data of both surgeries performed consecutively from scratch by the same thoracic surgeon and analyzing their learning curves.

## Materials and methods

### Patients

Perioperative data of mediastinal mass resection were retrospectively collected from 80 initial consecutive VATS cases from February 2018 to February 2020 and 80 initial consecutive RATS cases from March 2020 to March 2023. All the surgeries were done by a thoracic surgeon at the Affiliated Hospital of Xuzhou Medical University. Some of the clinical data were extracted from the hospital’s electronic files. This was a retrospective study and hence the need for informed consent from patients was waived off. The study was approved by the Ethics Committee of the Affiliated Hospital of Xuzhou Medical University (XYFY2023-KL291-01).

### Observed indicators

Basic clinical data including age, gender, body mass index, location of tumor, previous malignancy, comorbidities, smoking history, number of large mediastinal tumors, long and short tumor diameter and cost were collected. Perioperative observation indexes including approach, postoperative bleeding, conversion to open surgery, operation time (OT), pleural adhesion, volume of drainage, drainage time, length of hospital stay, postoperative hospital stay, and pathologic type were also collected.

### Preoperative preparation

Patients in both groups were given equal care before surgery. Preoperative routine examinations, such as electrocardiogram, cardiac color ultrasound, pulmonary function and chest enhancement computed tomography (CT), blood routine, biochemistry, coagulation, complete set of viruses, and blood type, were performed to clarify whether there were contraindications to surgery. Surgical treatment was performed after excluding relevant contraindications to surgery. If the CT indicated an enlarged intervertebral foramen at the tumor site and if an abnormal soft tissue shadow was seen in the spinal canal, further Magnetic Resonance Imaging (MRI) was required. If the MRI indicated a dumbbell-shaped posterior mediastinal tumor in the spinal canal, a neurological consult was required to determine the surgical procedure.

### Surgical technique

**Fig. 1:**
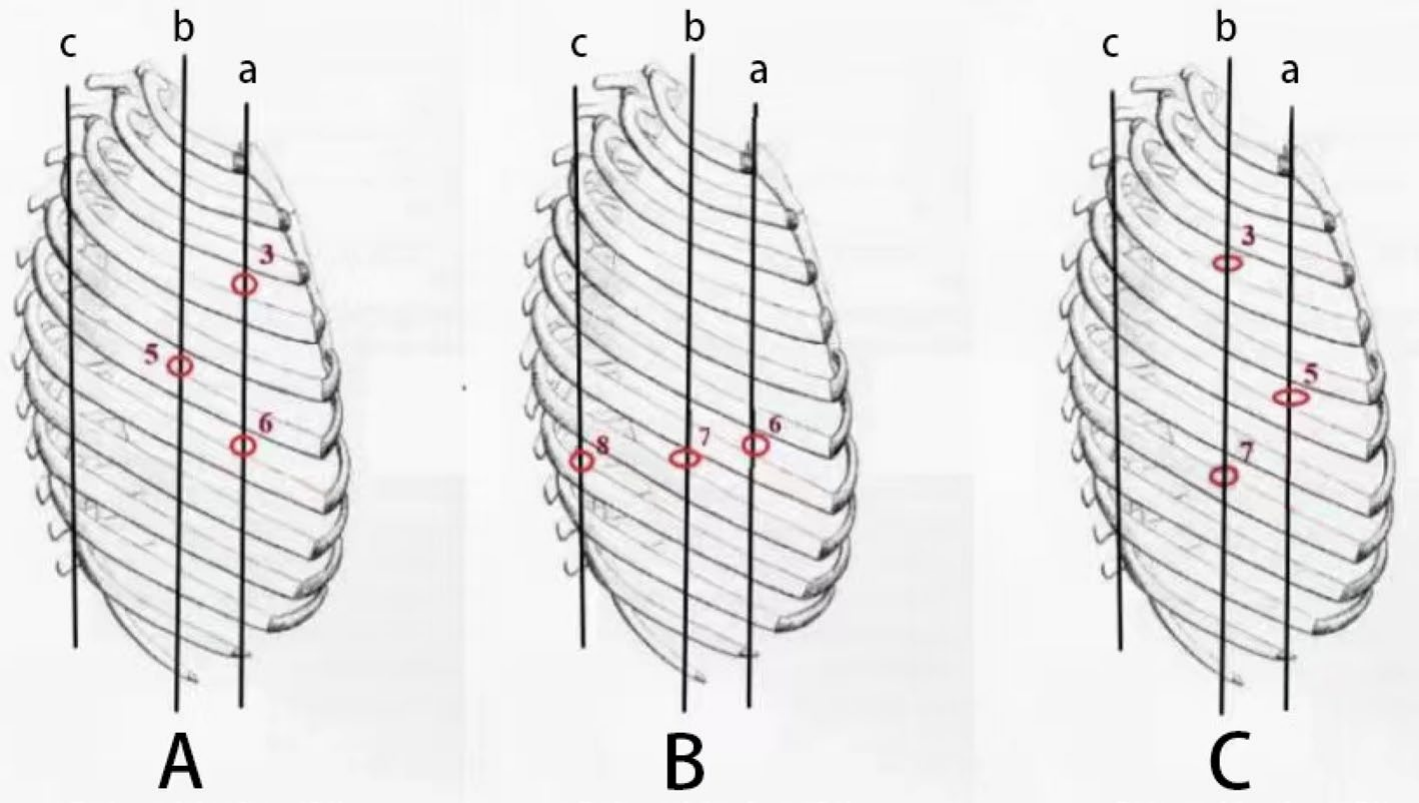
**A**–**C** represent the anterior robotic mediastinal, posterior superior mediastinal, and posterior inferior mediastinal approaches, respectively. a, b, and c represent the anterior axillary line, the mid-axillary line and the posterior axillary line, respectively. (This figure takes the right approach as an example. Middle mediastinal tumors can be selected from one of the above approaches according to the specific conditions.)

Da Vinci robotic surgery: after general anesthesia was successfully administered, the patient was intubated with a double-lumen endotracheal tube, a urinary catheter was left in place, and the healthy lung was ventilated. The tumor projection was outlined on the body surface based on CT, and the incision was designed based on the projection as required by the robotic manipulator arm (Fig. 1). For anterior mediastinal mass, the patient was placed in the supine position with the affected side of the chest elevated by 30 degrees. The incision was made in the “5-3-6” method. Five indicated the 5th intercostal space in the mid-axillary line and was used as the observation hole; 3 indicated the 1st operation hole. A perforated bipole was placed in the 3rd intercostal space in the anterior axillary line. Six indicated the 2nd operating hole in the 6th intercostal space in the anterior axillary line where the electric hook was placed. In the case of posterior superior mediastinal mass, the patient was laid on the healthy side, and the incision was made in the “7-8-6” method. Seven indicated the 7th intercostal space in the mid-axillary line that served as the observation hole; 8 indicated the 1st operation hole in the 8th intercostal space in the posterior axillary line, in which the perforated bipole was placed at the posterior line at the 8th intercostal space; and 6 was the 2nd operation hole at the 6th intercostal space in the anterior axillary line to place the electric hook. For posterior inferior mediastinal mass, the patient was laid on the healthy side, and the incision is made in the “5-3-7” method. Five indicated the observation hole at the 5th intercostal space in the anterior axillary line of the affected side; 3 indicated the operation hole for the 1st arm at the 3rd intercostal space in the mid-axillary line in which a perforated bipole was placed. Seven indicated the operating hole for the 2nd arm. An electric hook was placed in the mid-axillary line at the 7th intercostal space.

The Trocar microscope hole was connected to carbon dioxide (CO2) to establish an artificial pneumothorax (10 L/min flow of CO2 was continuously blown into the chest cavity to maintain 8 mmHg) to fully reveal the tissue structures.

*Anterior mediastinal mass with thymoma as an example* The location of the mass was explored, and the mediastinal pleura was opened through electric hook separation along the medial edge of the internal mammary vein, which was free cephalad to the internal mammary vein refluxing to the innominate vein. The thymus was separated along the pericardium and superior vena cava, and the left and right saphenous veins were seen to converge into the superior vena cava. The superior pole of the thymus was continued above the nameless vein, and the vessels emanating from the superior pole of the thymus were electrocoagulated using electric hooks. The thinner thymic veins were coagulated using bipolar electrocoagulation graspers, and the thicker thymic veins were dissected using vascular clamps. The thymus was continually freed downward and to the contralateral side to visualize the contralateral pleura and phrenic nerve and to ensure their safety and protection. The continued freeing was done until complete removal of both thymus glands. The operation was completed after checking for blood leakages, covering the wound with a hemostatic material, counting the instruments and gauze correctly, leaving one chest drain in place, and closing the incision layer by layer.

*Posterior mediastinal mass* The tumor was traced using a pair of non-invasive grasping forceps, and the mediastinal pleura was opened through electric hook electrocoagulation. The tumor was bluntly separated from outside the tumor envelope using a small gauze ball along the envelope. The operation was completed after checking for any blood leakages, covering the wound with a hemostatic material, counting the instruments and gauze, leaving one chest drain in place, and closing the incision layer by layer.

Thoracoscopic mediastinal mass resection surgery: The process is similar to the Da Vinci robot process and hence not described.

### Cumulative sum analysis

The cumulative sum (CUSUM) method was used to analyze operative times to determine the sum of the running differences between individual data points and the mean of all data points. *X*-axis included the oldest case to the newest case in a chronological order. We counted the difference between the individual operative time for each patient and the mean operative time for the series. The cumulative sum of these differences was calculated and represented graphically on the *Y*-axis and then a smooth curve was fitted based on the line graph [11, 12].

### Statistical analysis

Statistical analyses were performed using the Statistical Package for Social Sciences (SPSS) software version 25.0. Quantitative data were expressed as mean ± standard deviation (*x* ± *s*). Comparisons between groups were performed using the *t* test. Categorical variables were expressed as frequencies and percentages, and the Pearson chi-square test was used to test for differences between groups. If the theoretical frequency was less than 1, the Fisher exact probability method was used to test for differences between groups. The test level was *α* = 0.05 and *P* < 0.05 was considered statistically significant.

## Results

### Demographic and baseline characteristics of patients

The demographic and baseline characteristics of the two groups are shown in Table 1. The costs during hospitalization were statistically different between the two groups (*P* < 0.001). There were no statistically significant differences in terms of age, gender, BMI, tumor location, previous malignancy, comorbidities, smoking history, and long and short tumor diameter (*P* > 0.05).

**Table 1:** Demographic and baseline characteristics of patients

Characteristics	VATS (*n* = 80)	RATS (*n* = 80)	*P*-value
Age, years	50.39 ± 12.43	50.44 ± 13.40	0.981
Gender, *n*(%)			0.999
Male	32 (40)	32 (40)	
Female	48 (60)	48 (60)	
BMI, kg/m^2^	25.30 ± 3.32	25.14 ± 3.87	0.774
Location of tumor, *n*(%)			0.875
Anterior mediastinum	64 (80)	62 (77.5)	
Middle mediastinum	2 (2.5)	3 (3.8)	
Posterior mediastinum	14 (17.5)	15 (18.8)	
Previous malignancy, *n* (%)	5 (6.2)	9 (11.2)	0.401
Comorbidities, *n* (%)	18 (22.5)	16 (20.0)	0.847
Smoking history, *n* (%)	4 (5.0)	7 (8.8)	0.532
Number of large mediastinal tumors, *n* (%)	11 (13.75)	15 (18.75)	0.391
Tumor long diameter, mm	37.14 ± 21.88	40.10 ± 20.66	0.381
Tumor short diameter, mm	24.96 ± 14.10	29.40 ± 16.53	0.070
Cost, CNY	14,097.24 ± 7054.00	29,596.96 ± 12,260.85	< 0.001

### Perioperative information of patients

**Fig. 2:**
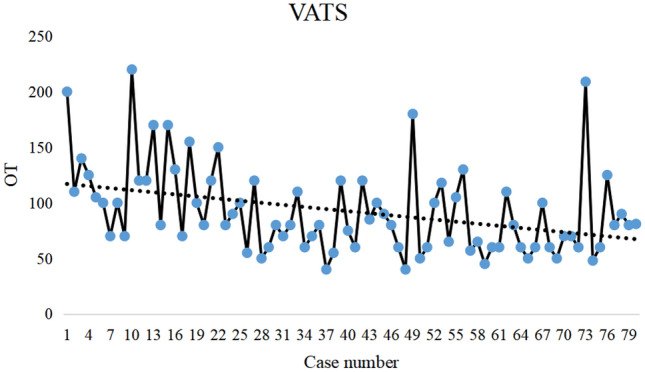
Changes in operation time for thoracoscopic mediastinal mass resection. The dash line shows an overall downward trend in operation time

**Fig. 3:**
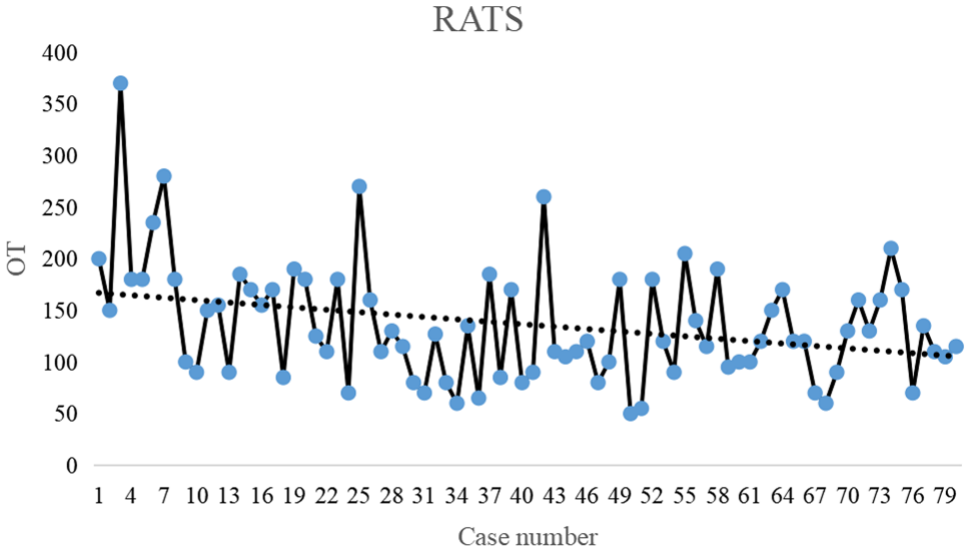
Changes in operation time for robotic mediastinal mass resection. The dash line shows an overall downward trend in operation time

All patients successfully completed the surgery. One patient in the VATS group was successfully treated conservatively for postoperative bleeding. One patient in the RATS group was transferred to open surgery due to complete pleural adhesions, and one patient underwent a second surgery for postoperative bleeding. There was no statistically significant difference between the RATS group and the VATS group in terms of the approach, pleural adhesions, volume of drainage, drainage time, and pathologic types (Table 2). However, the operative time in the RATS group (136.15 ± 57.08 min) was significantly longer than that in the VATS group (92.16 ± 39.48 min) and was statistically different (*P* < 0.001). According to the trend graphs of surgical time, we observed that the surgical time in the RATS group and the VATS group showed an overall decreasing trend (Figs. 2 and 3). The total hospital stay in the RATS group versus the VATS group (7.72 ± 3.06 d vs. 9.68 ± 4.07 d, *P* = 0.001) as well as the postoperative hospital stay (4.25 ± 1.42 d vs. 4.91 ± 1.94 d, *P* = 0.015) were significantly shorter.

**Table 2:** Perioperative information of patients

Characteristic	VATS (*n* = 80)	RATS (*n* = 80)	*P*-value
Approach, *n* (%)			0.999
Left	29 (36.3)	30 (37.6)	
Right	51 (63.7)	50 (62.5)	
Postoperative bleeding, *n *(%)	1 (1.3)	1 (1.3)	0.999
Conversion to open surgery, *n* (%)	0 (0)	1 (1.3)	0.999
Operation time (min), mean ± SD	92.16 ± 39.48	136.15 ± 57.08	< 0.001
Pleural adhesion, *n* (%)	13 (16.2)	9 (11.2)	0.491
Volume of drainage (ml), mean ± SD	265.38 ± 320.30	268.00 ± 304.27	0.958
Drainage time (d), mean ± SD	3.04 ± 1.42	2.83 ± 1.08	0.287
Length of hospital stay (days), mean ± SD	9.68 ± 4.07	7.72 ± 3.06	0.001
Postoperative hospital stay (d), mean ± SD	4.91 ± 1.94	4.25 ± 1.42	0.015
Pathologic types, *n* (%)			0.074
Thymoma	14 (17.5)	23 (28.7)	
Thymic hyperplasia	5 (6.2)	3 (3.8)	
Cysts	47 (58.8)	32 (40.0)	
Nerve sheath tumor	7 (8.8)	5 (6.2)	
Teratoma	2 (2.5)	5 (6.2)	
Other pathological types	5 (6.2)	12 (15.0)

### The results of the learning curve

**Fig. 4:**
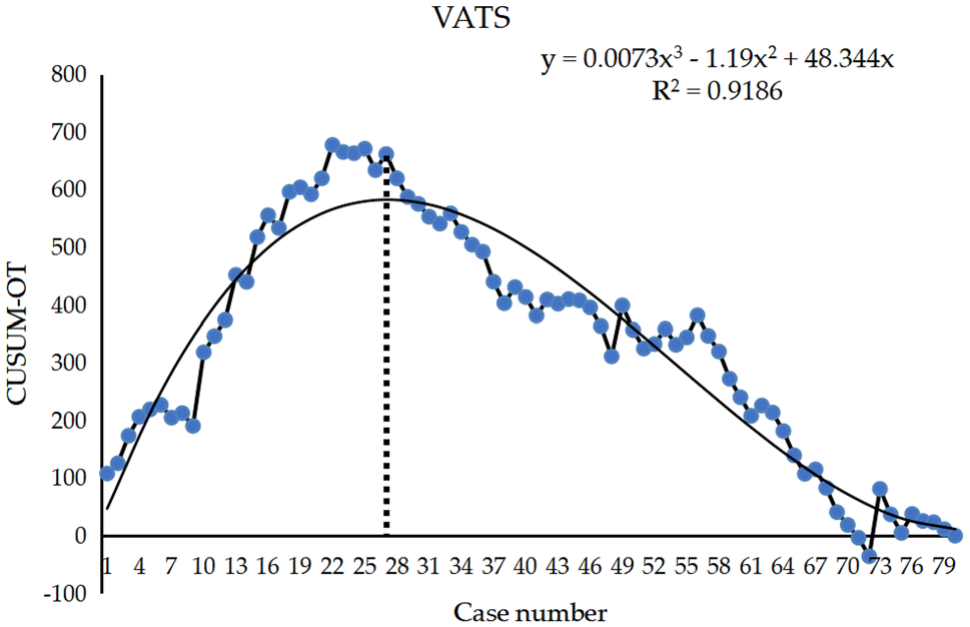
Scatter plot of CUSUM learning curve for VATS resection. The smoothed curve is a fitted model of this learning curve, and the vertical dashed line shows that the apex of the curve corresponds to the horizontal coordinates of the 27th case

**Fig. 5:**
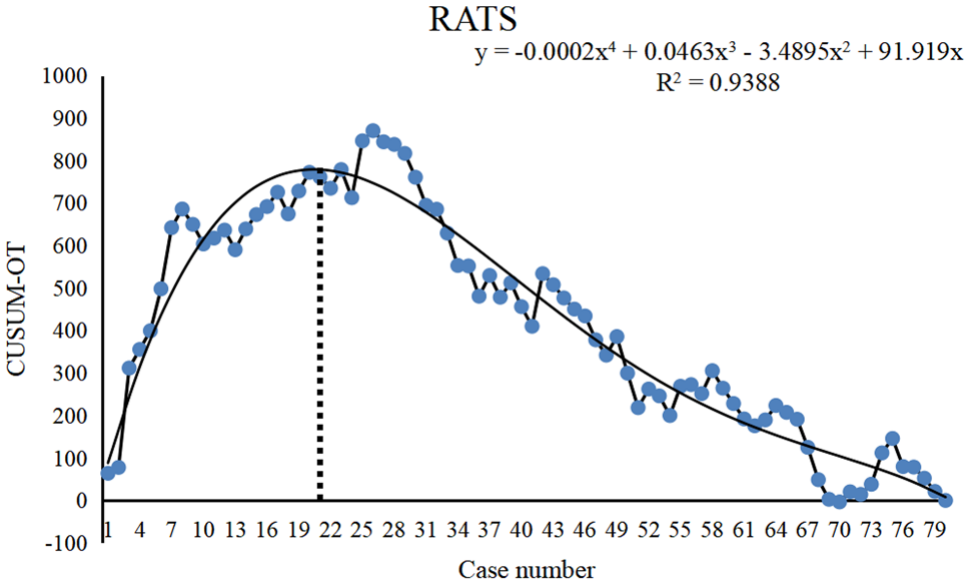
Scatter plot of CUSUM learning curve for RATS resection. The smoothed curve is a fitted model of this learning curve, and the vertical dashed line shows that the apex of the curve corresponds to the horizontal coordinates of the 21th case

CUSUM charts for the time of operation showed the learning curves for the VATS and RATS groups (Figs. 4 and 5). The fitted model for the VATS group was a 3D curve with the equation CUSUM = 0.0073*X*^3^ − 1.19*X*^2^ + 48.344*X* (*X* is the number of surgical cases), and the fitted model for the RATS group was a 4D curve with the equation CUSUM =− 0.0002*X*^4^ + 0.0463X^3^ − 3.4895*X*^2^ + 91.919*X* (*X* is the number of surgical cases). According to the equations fitted above, the learning curves of both VATS and RATS were divided into two phases. The first phase was the initial learning period or the novice phase, which included the first 27 cases in the VATS group and the first 21 cases in the RATS group. The second phase was the proficiency period, which represented the surgeon’s proficiency level in the surgery and included the latter 53 cases in the VATS group and the 59 cases in the RATS group.

### Comparison of perioperative outcomes between learning and mastery periods

We then compared the learning and mastery (proficiency) periods in the VATS and RATS groups (Table 3). The operative times in the learning and mastery periods in the VATS group were 116.67 ± 40.71 min and 79.68 ± 32.68 min, respectively. In the RATS group, the operative times in the learning and mastery periods were 172.38 ± 65.70 min and 123.25 ± 48.00 min, respectively. The operative time in the learning phase was significantly longer than that in the mastery phase when both groups were independently compared between groups (*P* < 0.001). We also found that the learning period was longer than the mastery period when comparing the total hospitalization time and postoperative hospitalization time in the VATS and RATS groups. Interestingly, the drainage time during the mastery period was significantly shorter than that during the learning period in the VATS group. However, we did not find the same phenomenon in the RATS group. The other indicators, such as the path of entry into the chest, intraoperative adhesions, drainage volume, and type of pathology were not statistically significant in the learning period versus the mastery period.

**Table 3:** Outcome information for learning and mastery periods

Characteristic	VATS			RATS		
	Learning period (*n* = 27)	Mastery period (*n* = 53)	*P*	Learning period (*n* = 21)	Mastery period (*n* = 59)	*P*
Approach, *n* (%)			0.999			0.076
Left	10 (37.0)	19 (35.8)		4 (19.0)	26 (44.1)	
Right	17 (63.0)	34 (64.2)		17 (81.0)	33 (55.9)	
Postoperative bleeding, *n* (%)	1 (3.7)	0 (0)	0.729	1 (4.8)	0 (0)	0.587
Conversion to open surgery, *n* (%)	0 (0)	0 (0)	0.999	1 (4.8)	0 (0)	0.587
Operation time, min	116.67 ± 40.71	79.68 ± 32.68	< 0.001	172.38 ± 65.70	123.25 ± 48.00	< 0.001
Pleural adhesion, *n* (%)	5(18.5)	8(15.1)	0.943	4(19.0)	5(8.5)	0.360
Volume of drainage, ml	303.70 ± 234.54	245.85 ± 356.65	0.448	246.19 ± 172.27	275.76 ± 340.05	0.705
Drainage time, d	3.85 ± 1.70	2.62 ± 1.04	< 0.001	2.90 ± 0.94	2.80 ± 1.13	0.695
Length of hospital stay, days	12.67 ± 4.51	8.15 ± 2.82	< 0.001	10.43 ± 3.80	6.76 ± 2.04	< 0.001
Postoperative hospital stay, d	6.48 ± 2.14	4.11 ± 1.24	< 0.001	5.00 ± 1.92	3.98 ± 1.09	0.004
Pathologic types, *n* (%)			0.540			0.164
Thymoma	4 (14.8)	10 (18.9)		5 (23.8)	18 (30.5)	
Thymic hyperplasia	0 (0.0)	5 (9.4)		2 (9.5)	1 (1.7)	
Cysts	18 (66.7)	29 (54.7)		9 (42.9)	23 (39.0)	
Nerve sheath tumor	3 (11.1)	4 (7.5)		0 (0.0)	5 (8.5)	
Teratoma	1 (3.7)	1 (1.9)		0 (0.0)	5 (8.5)	
Other pathological types	1 (3.7)	4 (7.5)		5 (23.8)	7 (11.9)	

## Discussion

In recent years, robotic systems for surgery have advanced in China. Compared with open surgery and VATS, robotic surgery has unique advantages in the resection of mediastinal lesions, including high-definition 3D vision, an interactive robotic arm, internal wrist system, and a tremor filter. Mediastinal lesions, especially anterior mediastinal masses, are the ideal indication for robotic surgery [13]. Using cumulative and analytical methods, we determined the learning curves for objective and measurable outcomes of thoracoscopic and robotic-assisted mediastinal mass resection, respectively. Combined with extensive thoracoscopic experience on the part of the surgeon and the many advantages of the robotic system, the learning curve for RATS resection is shorter than that for VATS resection.

We found that mediastinal mass resection using RATS required 21 procedures to attain the mastery stage. Kamel MK et al. found that mediastinal mass resection required approximately 15 to 20 procedures to overcome the learning curve of the da Vinci robot to attain proficiency [14], which is similar to our results. However, thoracoscopic mediastinal mass resection requires a cumulative experience of 27 procedures to overcome the initial learning stage and attain proficiency. Combined with a more complex method of operation of the robotic system, it suggests that a certain amount of thoracoscopic experience can shorten the learning curve of robot-assisted mediastinal mass resection. This result is also in line with our pre-study conjecture that surgeons performing robotic surgery are well versed in the anatomy, surgical steps, and critical site freeing processes associated with mediastinal mass, whereas newer surgeons training for thoracoscopy need to be cautious about these processes. Unlike reports by Gómez-Hernández MT et al. in the field of lung lobes, they did not find any differences when comparing the learning curves of robotics and thoracoscopy [15]. This variability may be because the thoracic cavity has a large space, which makes robotics and thoracoscopy difficult to operate on to the same extent. However, mediastinal tumors are operated in a more confined space, which limits thoracoscopy but robot-assisted procedures are more comfortable and convenient to operate in the confined space of the mediastinum.

We divided the VATS and RATS groups into learning and mastery periods based on the learning curve and compared them. The operation time and postoperative hospital stay in the mastery phase was shorter than that in the learning phase, which means that with more experience, the surgeon could better control the parameters of the surgery and handle challenges during surgery with greater ease. We similarly compared the cost to the patient, which inevitably increases for patients undergoing robotic surgery. Despite the advantages that surgeons have in using robotic systems in mediastinal tumor resection, some patients choose thoracoscopic surgery because of its relatively low cost. However, thoracoscopy is often difficult for surgeons to operate on tumors that are deep and have a narrow operating space. The resection of thoracic parietal tumors in the early stage through thoracoscopy was extremely challenging because of its proximity to the subclavian artery, the high number of peripheral nerves, and the relative fixation of the tumor. The thoracoscope was less flexible and did not allow for easy observation and exposure, which in turn increased the potential risk to the patient. Since the introduction of the da Vinci robotic system in our center, the flexible robotic arm led to better surgical field of view and excellent operating angle. Complete tumor resection has been possible, which further reduces risk to patient and ensures safety during surgery.

In this study, all patients were successfully operated on, including one patient in the RATS group who was converted to open surgery due to complete pleural adhesions, and one patient who underwent a second surgery due to postoperative bleeding. We achieved good perioperative outcomes in terms of postoperative complications, operative time, drainage time, postoperative hospital stay, and resection of large mediastinal tumors, demonstrating the safety and effectiveness of robotic-assisted mediastinal surgery. This is consistent with the findings reported by Savitt et al. and Alvarado et al. [16, 17]. In our study, large mediastinal tumors (> 6 cm) were seen in 11 cases in the VATS group and 15 in the RATS group, all of which were completely resected. Seong YW et al. reported that the use of the da Vinci robot showed good early clinical outcomes in patients with relatively small mediastinal masses compared to a matched group of patients who underwent open surgery [18]. However, this study did not address the large mediastinal tumors, probably because the limited retrosternal space and adjacent vital structures make complete resection of mediastinal masses challenging. For large mediastinal tumors, it was previously believed that the traditional open approach provides a good operative field and facilitates combined resection and reconstruction of the invaded vital structures, thereby better meeting safety and oncologic principles. However, Jiang B et al. demonstrated that RATS is safe and effective for the resection of large anterior mediastinal tumors and that cordectomy with RATS is technically feasible compared with VATS and open surgery [19]. Our study similarly demonstrated that complete resection of large mediastinal tumors was also achievable using the da Vinci robotic system.

In this study, the postoperative hospital stay of the patients in the VATS group was significantly shorter than those in the RATS group, which is the same as the results reported by Shen et al. in a meta-analysis [20]. The mean operative time of the patients in the RATS group was 136.15 ± 57.08 min, which was longer than that of the patients in the VATS group, 92.16 ± 39.48 min. We believe that this is due to the following two reasons: (1) after general anesthesia, the surgical assistants require additional time to connect the robotic arm of the da Vinci robot and the camera system and disconnect the relevant connections at the end of the operation; (2) the surgeon in charge and the assistants are not familiar with the surgical system at the initial stage, and there is a lack of tacit understanding with the nursing and anesthesiology teams.

This study also has some limitations. First, as a retrospective study, it was susceptible to selection bias. Second, the surgeon had an experience of more than 50 thoracoscopic lung surgeries at the time of thoracoscopic mediastinal tumor resection, whereas the operator had an experience of only one robotic lung surgery at the time of initiating robotic mediastinal tumor resection. Third, the surgical assistants varied during thoracoscopic surgery.

Finally, according to our study, RATS resection has a shorter learning curve compared to VATS resection. We hope that in the future, we can establish a program to accelerate the learning curve for young surgeons performing RATS, thus reducing the potential risks to patients during the learning period.

## Data Availability

Data available upon request.

## References

[1] Ghigna MR et al (2021) Mediastinal tumours and pseudo-tumours: a comprehensive review with emphasis on multidisciplinary approach. Eur Respir Rev 30(162). 10.1183/16000617.0309-2020 PMC9488622 34615701

[2] Wightman SC et al (2019) Non-myasthenia gravis immune syndromes and the thymus: is there a role for thymectomy? Thorac Surg Clin 29(2):215–225 30928003 10.1016/j.thorsurg.2018.12.008

[3] Issoufou I et al (2016) Neurogenic mediastinal tumors in adults. Rev Pneumol Clin 72(5):310–315 27567980 10.1016/j.pneumo.2016.05.002

[4] Wang GW et al (2019) Comparison between thoracoscopic and open approaches in thymoma resection. J Thorac Dis 11(10):4159–4168 31737299 10.21037/jtd.2019.09.85 PMC6837992

[5] Straughan DM et al (2015) Robotic-assisted videothoracoscopic mediastinal surgery. Cancer Control 22(3):326–330 26351888 10.1177/107327481502200310

[6] Guo C et al (2016) Video-assisted thoracic surgery compared with posterolateral thoracotomy for mediastinal bronchogenic cysts in adult patients. J Thorac Dis 8(9):2504–2511 27747002 10.21037/jtd.2016.08.29 PMC5059344

[7] Fang Y et al (2021) Video-assisted thoracoscopic surgery is safe and reliable for large and invasive primary mediastinal tumors. Wideochir Inne Tech Maloinwazyjne 16(1):163–168 33786130 10.5114/wiitm.2020.94528 PMC7991948

[8] Chen K et al (2020) Robot-assisted thoracoscopic surgery for mediastinal masses: a single-institution experience. J Thorac Dis 12(2):105–113 32190360 10.21037/jtd.2019.08.105 PMC7061195

[9] Li XK et al (2020) Clinical efficacy of robot-assisted thoracoscopic surgery for posterior mediastinal neurogenic tumors. J Thorac Dis 12(6):3065–3072 32642229 10.21037/jtd-20-286 PMC7330773

[10] Geraci TC et al (2021) Beyond the learning curve: a review of complex cases in robotic thoracic surgery. J Thorac Dis 13(10):6129–6140 34795964 10.21037/jtd-2019-rts-05 PMC8575821

[11] Biswas P et al (2008) A risk-adjusted CUSUM in continuous time based on the Cox model. Stat Med 27(17):3382–3406 18288785 10.1002/sim.3216

[12] Kim NR et al (2022) Comparison of surgical outcomes and learning curve for robotic versus laparoscopic living donor hepatectomy: a retrospective cohort study. Int J Surg 108:107000 36379423 10.1016/j.ijsu.2022.107000

[13] Li H et al (2018) Robotic-assisted mediastinal surgery: the first Chinese series of 167 consecutive cases. J Thorac Dis 10(5):2876–2880 29997952 10.21037/jtd.2018.04.138 PMC6006102

[14] Kamel MK et al (2017) Robotic thymectomy: learning curve and associated perioperative outcomes. J Laparoendosc Adv Surg Tech A 27(7):685–690 28121481 10.1089/lap.2016.0553

[15] Gomez-Hernandez MT et al (2022) The robotic surgery learning curve of a surgeon experienced in video-assisted thoracoscopic surgery compared with his own video-assisted thoracoscopic surgery learning curve for anatomical lung resections. Eur J Cardiothorac Surg 61(2):289–296 34535994 10.1093/ejcts/ezab385

[16] Savitt MA et al (2005) Application of robotic-assisted techniques to the surgical evaluation and treatment of the anterior mediastinum. Ann Thorac Surg 79(2):450–455 (**455**) 15680812 10.1016/j.athoracsur.2004.07.022

[17] Alvarado CE et al (2022) Robotic approach has improved outcomes for minimally invasive resection of mediastinal tumors. Ann Thorac Surg 113(6):1853–1858 34217691 10.1016/j.athoracsur.2021.05.090

[18] Seong YW et al (2014) Early clinical outcomes of robot-assisted surgery for anterior mediastinal mass: its superiority over a conventional sternotomy approach evaluated by propensity score matching. Eur J Cardiothorac Surg 45(3):e68–e73 (**e73**) 24321994 10.1093/ejcts/ezt557

[19] Jiang B et al (2023) Robot-assisted thymectomy in large anterior mediastinal tumors: a comparative study with video-assisted thymectomy and open surgery. Thorac Cancer 14(3):267–273 36433677 10.1111/1759-7714.14744 PMC9870738

[20] Shen C et al (2022) Robot-assisted thoracic surgery versus video-assisted thoracic surgery for treatment of patients with thymoma: a systematic review and meta-analysis. Thorac Cancer 13(2):151–161 34806328 10.1111/1759-7714.14234 PMC8758429

